# Endogenous glutamine is rate-limiting for anti-CD3 and anti-CD28 induced CD4^+^ T-cell proliferation and glycolytic activity under hypoxia and normoxia

**DOI:** 10.1042/BCJ20220144

**Published:** 2022-06-13

**Authors:** Jonas A. Wik, Azazul Chowdhury, Shrikant Kolan, Nasser E. Bastani, Gaoyang Li, Kazi Alam, Franco Grimolizzi, Bjørn S. Skålhegg

**Affiliations:** 1 Division of Molecular Nutrition, Department of Nutrition, Institute of Basic Medical Sciences, University of Oslo, Oslo, Norway; 2 Department of Pathology, Institute of Clinical Medicine, University of Oslo, Oslo, Norway

**Keywords:** BPTES, CD4^+^ T cells, glutamine, hypoxia, metabolism, normoxia

## Abstract

To meet the demand for energy and biomass, T lymphocytes (T cells) activated to proliferation and clonal expansion, require uptake and metabolism of glucose (Gluc) and the amino acid (AA) glutamine (Gln). Whereas exogenous Gln is converted to glutamate (Glu) by glutaminase (GLS), Gln is also synthesized from the endogenous pool of AA through Glu and activity of glutamine synthase (GS). Most of this knowledge comes from studies on cell cultures under ambient oxygen conditions (normoxia, 21% O_2_). However, *in vivo*, antigen induced T-cell activation often occurs under moderately hypoxic (1–4% O_2_) conditions and at various levels of exogenous nutrients. Here, CD4^+^ T cells were stimulated for 72 h with antibodies targeting the CD3 and CD28 markers at normoxia and hypoxia (1% O_2_). This was done in the presence and absence of the GLS and GS inhibitors, Bis-2-(5-phenylacetamido-1,3,4-thiadiazol-2-yl) ethyl sulfide (BPTES) and methionine sulfoximine (MSO) and at various combinations of exogenous Gluc, Gln and pyruvate (Pyr) for the last 12 h of stimulation. We found that T-cell proliferation, viability and levels of endogenous AA were significantly influenced by the availability of exogenous Gln, Gluc and Pyr as well as inhibition of GLS and GS. Moreover, inhibition of GLS and GS and levels of oxygen differentially influenced oxygen consumption rate (OCR) and extracellular acidification rate (ECAR). Finally, BPTES-dependent down-regulation of ECAR was associated with reduced hexokinase (HK) activity at both normoxia and hypoxia. Our results demonstrate that Gln availability and metabolism is rate-limiting for CD4^+^ T-cell activity.

## Introduction

Antigen recognition and co-stimulation of CD4^+^ T cells to proliferation, differentiation and clonal expansion *in vivo* are metabolically challenging processes as availability of key nutrients may vary [1]. In addition, many tissues and organs including spleen, thymus and lymphatic fluids experience reduced levels of O_2_ often referred to as hypoxia which under physiological conditions is O_2_ tensions <2 kPa or <4% O_2_ [2–4]. This suggests that *in vivo* T-cell activation must occur under both varying levels of nutrients as well as O_2_ (≥1 ≤ 21% O_2_). To meet the demand for energy and biomass, activated CD4^+^ T cells are fully dependent on rapid uptake and consumption of glucose (Gluc) and glutamine (Gln). This occurs by up-regulating the Gluc transporter 1 (GLUT1) and the Gln transporter, alanine (Ala) serine (Ser) cysteine (Cys) transporter 2 (ASCT2) [5,6]. Uptake is followed by increased Gluc and Gln combustion ultimately leading to the production and secretion of lactate (Lac) and Ala [7,8]. Lac is produced from pyruvate (Pyr) derived from Gluc in the glycolytic pathway by Lac dehydrogenase (LDH) [9]. Pyr may also be used as a substrate to produce Ala, in a reaction regulated by Ala transaminase (ALT). Here ALT regulates transamination of Pyr by transfer of an amino group from Gln-derived glutamate (Glu) to produce Ala and alpha-ketoglutarate (α-KG) [10]. Glycolytic Lac production and Gln consumption in rapidly proliferating cells is referred to as the Warburg effect and Gln addiction, respectively. The Warburg effect and Gln addiction occurs at both normoxia (21% O_2_) and hypoxia (1–4% O_2_). Whereas the first step in glycolysis is phosphorylation of Gluc to produce Gluc 6-phosphate (G6P) by hexokinase (HK) [11], the first step in Gln metabolism is the conversion of Gln to Glu by mitochondrial glutaminase (GLS) [12,13]. Endogenously produced Glu has several fates in addition to protein synthesis. It may act as a substrate for nucleotide-, fatty acid-, hexosamine-, non-essential AA-, and Nicotinamide adenine dinucleotide phosphate (NADPH) synthesis and metabolism. During its metabolism Glu may enter various metabolic pathways, including the pentose phosphate pathway (PPP) and the tricarboxylic acid (TCA) cycle. In the latter case as α-KG [13–17]. Finally, in the cytoplasm, Glu may be converted back to Gln by Gln synthetase (GS) in an ATP dependent manner [18–21]. While GLS and GS are both present across different cell- and tissues types, most tissues can be grouped into Gln consumers or Gln producers [19]. Furthermore, whereas the GLS1 isoform glutaminase C (GAC) is localized in the mitochondria and is up-regulated in CD4^+^ T cells activated by anti-CD3/CD28 at normoxia, but not hypoxia, GS is localized in the cytoplasm and is up-regulated under hypoxia, but not normoxia [22]. This suggests an oxygen-dependent regulation of Gln metabolism.

Alterations in the levels of extracellular nutrients regulate the metabolic state of various cells. E.g. extracellular Gln deprivation has been shown to metabolically reprogram AA metabolism that can be partially compensated by exogenous aspartate (Asp), but not asparagine (Asn) [14–16,23]. This is due to a lack of functional asparaginase, the enzyme required to deamidate Asn to Asp, in most mammalian cells [16,24]. Because extracellular Asn can support protein synthesis and proliferation in Gln-deprived cells, Asn can be considered a conditionally essential amino acid, despite not contributing to the TCA cycle [14,16]. Moreover, elevated levels of arginine (Arg) a precursor for endogenous Glu induces global metabolic changes in T cells promoting central memory like T cells with improved capacity of survival [25]. Elevated levels of endogenous Arg is further associated with metabolic shift from glycolysis to oxidative phosphorylation (OXPHOS) [25]. In CD4^+^ T cells Arg can also be metabolized to form ornithine which can to some extent support proline (Pro) synthesis [25]. Finally, it has been shown that the levels of several endogenous AAs, including Gln, Ser and Asn dictate glucose uptake and aerobic glycolysis [26–30]. Together, this suggests that endogenous availability of AAs and O_2_ levels are key mediators of metabolic reprogramming important for T-cell activation, differentiation and proliferation.

We have tested the effect of O_2_, nutrient availability on anti-CD3/CD28-induced human CD4^+^ T-cell proliferation, cell viability, and ATP production. We find that both endogenous and exogenous availability and metabolism of Gln are rate-limiting factors for CD4^+^ T-cell proliferation and viability. Moreover, inhibition of Gln catabolism but not anabolism down-regulate glycolytic activity both at normoxia and hypoxia. Our results implicate Gln as crucial for levels of endogenous AA and glycolytic activity and hence, as rate-limiting for CD4^+^ T-cell proliferation and activity irrespective of normoxic and hypoxic condition.

## Results

### Endogenous glutamine availability for anti-CD3/CD28 induced CD4^+^ T-cell proliferation is vital irrespective of oxygen availability

To test sensitivity to Gln deprivation for CD4^+^ T-cell proliferation and viability at ambient and hypoxic O_2_ levels, we stimulated CD4^+^ T cells with anti-CD3/CD28 for 72 hours at 20% (normoxia) and 1% (hypoxia) O_2_.

**Figure 1.**
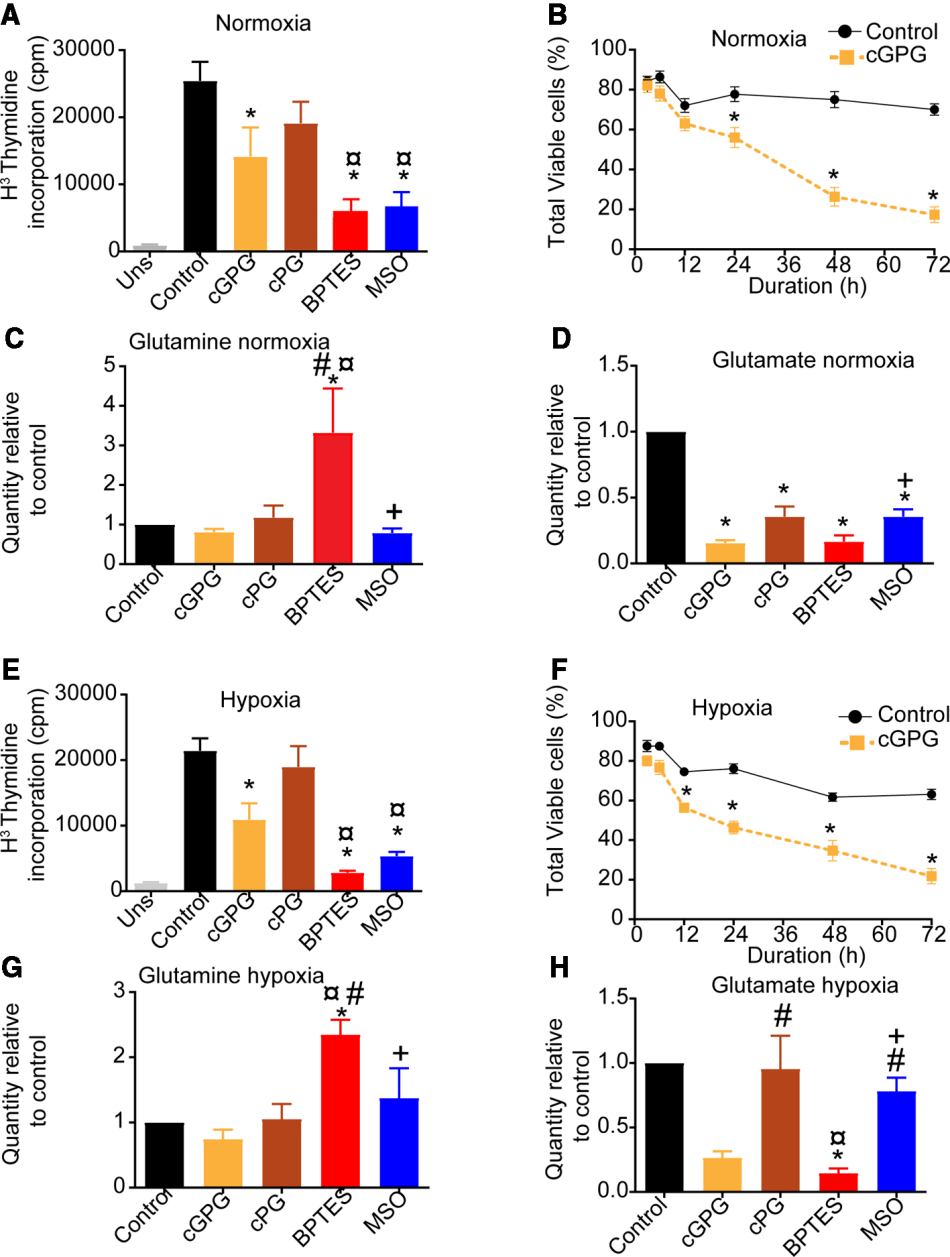
Gln deprivation reduces proliferation and viability of CD4^+^ T cells. (**A**) Thymidine incorporation rate in anti-CD3/CD28-stimulated CD4^+^ T cells under normoxia (21% O_2_) at 72 h post anti-CD3/CD28-stimulation following in complete media (control) or after deprivation of Gluc, Pyr and Gln (cGPG), Gluc and Pyr (cPG) or treatment with BPTES (25 µM) or MSO (7.5 µM) for the last 12 h. (**B**) Viability of αCD3/CD28-stimulated CD4^+^ T cells in control or following cGPG depletion at normoxia (21% O_2_). Relative levels of Gln (**C**) and Glu (**D**) at 72 h post anti-CD3/CD28-stimulation following 12 h of cGPG, cGP depletion, or BPTES or MSO treatment at normoxia (21% O_2_). (**E**) Thymidine incorporation rate of αCD3/CD28-stimulated CD4^+^ T cells under hypoxia (1% O_2_) at 72 h following either cGPG, cPG depletion or treatment with BPTES or MSO for the last 12 h. (**F**) Viability of αCD3/CD28)-stimulated CD4^+^ T cells in control or following cGPG depletion at hypoxia (1% O_2_). Relative levels of Gln (**G**) and Glu (**H**) at 72 h following 12 h of cGPG, cGP depletion, or BPTES or MSO treatment at hypoxia (1% O_2_). Data are mean ± SEM of three independent experiments in triplicates. * *P* < 0.05 compared with control , # *p* < 0.05 compared to cGPG, ¤ *P* < 0.05 compared to cPG, + *P* < 0.05 compared with BPTES.

This was done in the presence and absence of the GLS and GS inhibitors, BPTES (25 µM) and MSO (7.5 mM), respectively, and at various combinations of exogenous Gluc, Gln and Pyr deprivation for the last 12 h of stimulation. This revealed that at normoxia, depleting extracellular Gln, Glc and Pyr (cGPG), but not Pyr and Gluc (cPG), significantly reduced proliferation (Figure 1A). Blocking Gln anaplerosis or synthesis using BPTES or MSO, respectively, also significantly reduced proliferation (Figure 1A). This is in line with our previous observations that GLS inhibition reduces proliferation without inducing apoptosis or necrosis [22]. Viability over 72 h was also significantly reduced upon cGPG depletion at normoxia (Figure 1B). Relative quantification of endogenous Gln revealed that its level was unaffected by cGPG and cPG, depletion, respectively, and after MSO treatment. Moreover, endogenous Gln levels increased in the presence of BPTES (Figure 1C). In contrast with this, the endogenous concentration of Glu was significantly decreased after cGPG and cPG, depletion and by the BPTES and MSO treatments (Figure 1D). Consistent with previous reports glutathione (GSH) concentrations were dependent on Gln and its level reduced upon cGPG depletion and after BPTES and MSO treatment. It should be noted that cPG depletion did not affect endogenous GSH levels (Supplementary Figure S1A) [31].

At hypoxia, proliferation was significantly reduced upon cGPG depletion as well as after treatment with BPTES and MSO, but not by cPG depletion (Figure 1E). This was similar to what was observed at normoxia for the same conditions. Furthermore, cGPG depletion significantly reduced viability over 72 h (Figure 1F). Moreover, whereas BPTES treatment at hypoxia resulted in accumulation of endogenous Gln, depleting cGPG and cPG or inhibiting GS did not affect endogenous Gln levels (Figure 1G). Furthermore, endogenous levels of Glu at hypoxia were reduced upon cGPG depletion and BPTES treatment, but not by cPG depletion or MSO treatment, contrasting the effects observed at normoxia (Figure 1H). Again, GSH concentrations were depleted upon cGPG depletion as well as after BPTES and MSO treatment, however, in contrast with normoxia, GSH was also depleted upon cPG depletion at hypoxia. As seen in Figure 1, cGPG and cPG depletion or inhibition of either GLS or GS differentially influenced endogenous Gln and Glu levels at normoxia and hypoxia, with no clear correlation in relative abundance of Gln or Glu.

### Gln availability, GLS and GS activity differentially affects oxygen consumption at normoxia and hypoxia

**Figure 2.**
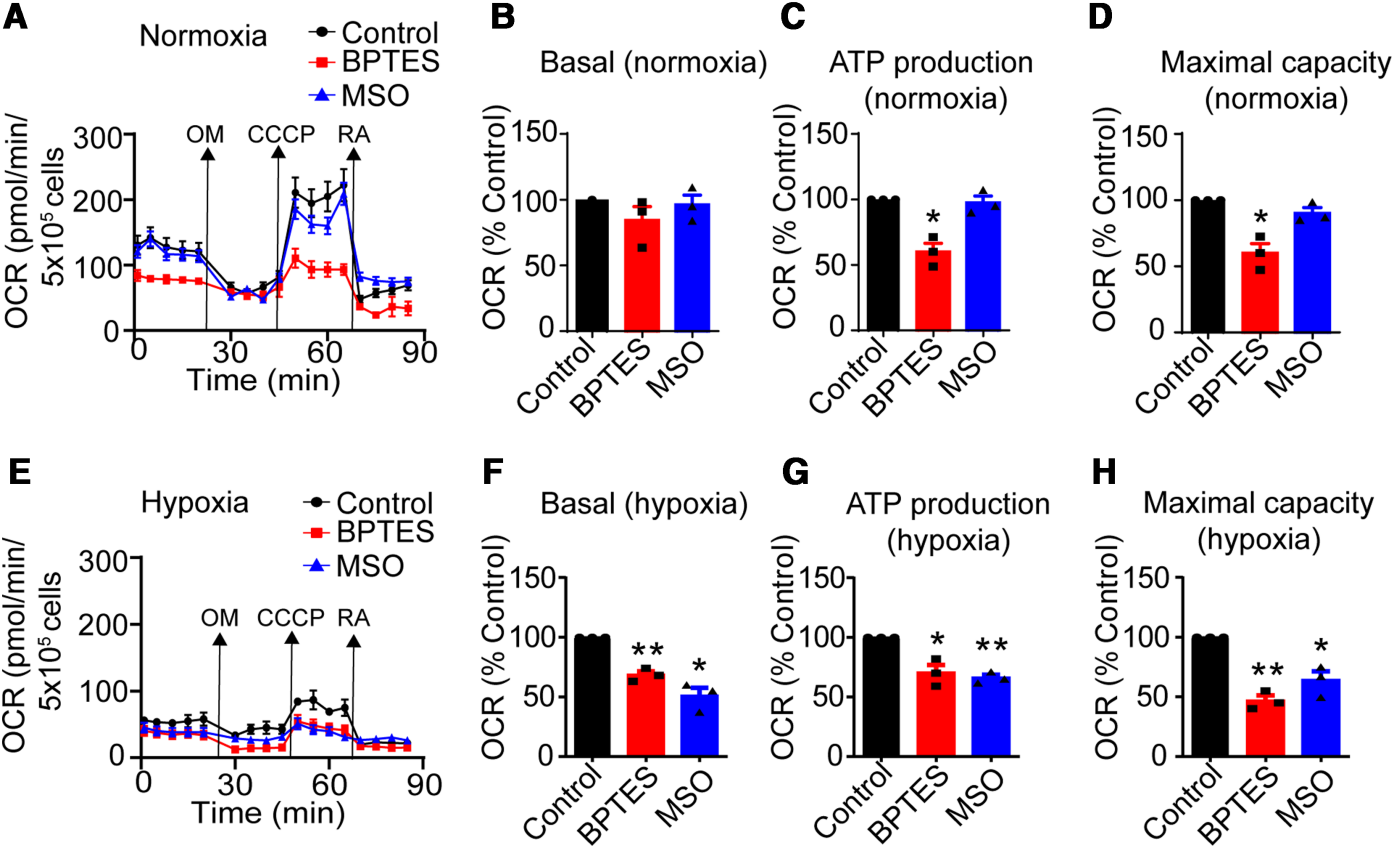
Inhibition of Gln metabolism differentially affects OCR at normoxia and hypoxia. (**A**) Representative Seahorse experiment of control (●), BPTES stimulated (▪) and MSO (▴) treated CD4^+^ T cells at normoxia. Basal (**B**), ATP production (**C**) and maximal OCR (**D**) of BPTES and MSO treated CD4^+^ T cells at normoxia relative to control. (**E**) Representative Seahorse experiment of control (●), BPTES stimulated (▪) and MSO (▴) treated cells at hypoxia. Basal (**F**), ATP production (**G**) and maximal OCR (**H**) of BPTES and MSO treated CD4^+^ T cells relative to control at hypoxia. Data are mean ± SEM of three independent experiments in triplicates. * *P* < 0.05 compared with control, ** *P* < 0.01 compared with control.

The results above imply that nutrient restriction, oxygen availability and manipulation of GLS and GS activity influence CD4^+^ T-cell proliferation and viability by mechanisms involving Gln availability. Through the TCA cycle Gln supports nicotinamide dinucleotide (NADH) and flavin adenosine dinucleotide (FADH) production and carbon residues required for cell proliferation and growth [32,33]. To investigate the role of Gln and O_2_ in regulating CD4^+^ T-cell proliferation OCR was next measured in the presence and absence of BPTES and MSO at normoxia and hypoxia (see material and methods for details). This shows that at normoxia ATP production as well as maximum OCR were significantly reduced in the presence of BPTES but not MSO, whereas basal OCR was not affected by either BPTES or MSO (Figure 2A–D). In contrast, at hypoxia both BPTES and MSO effects were associated with reduced, basal OCR, ATP production and maximal OCR (Figure 2E–H). Together this implies that at hypoxia, but not normoxia, GS activity is required for optimal T-cell activity.

### Gln availability, GLS and GS activity differentially affects the pool of endogenous amino acids

**Figure 3.**
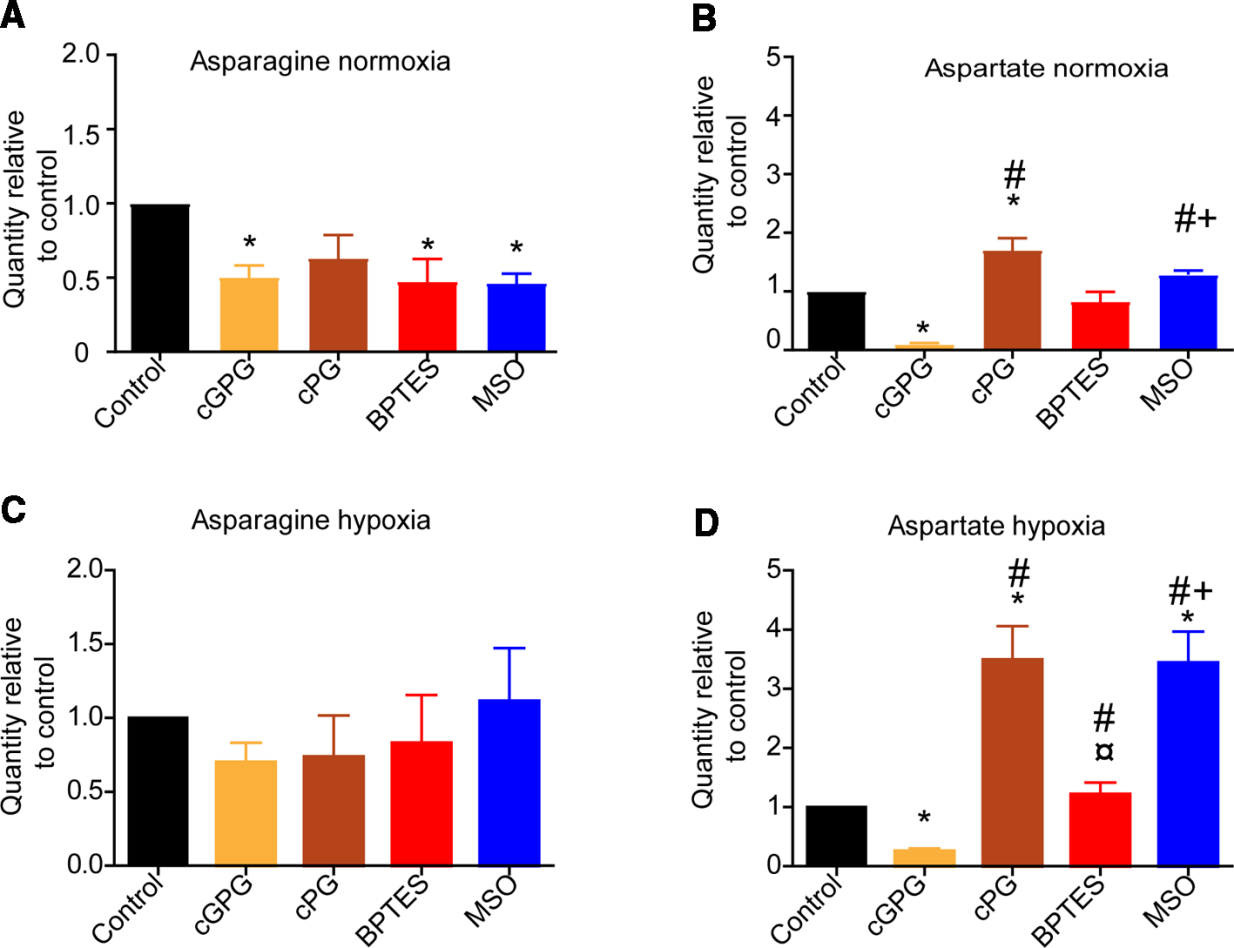
Gln deprivation and inhibition of Gln metabolism differentially affects Asn and Asp at normoxia and hypoxia. Relative quantities of Asn (**A**) and Asp (**B**) at normoxia and relative quantities of Asn (**C**) and Asp (**D**) at hypoxia relative to control at 72 h following 12 h of cGPG, cPG depletion, or BPTES or MSO treatment. Data are mean ± SEM of three independent experiments in triplicates. * *P* compared with control # *P* < 0.05 compared with control, ¤ *P* < 0.05, compared with cPG, + *P* < 0.05 compared with BPTES.

As seen in Figure 1, cGPG depletion or inhibition of either GLS or GS differentially influenced endogenous Gln and Glu levels, with no clear correlation in Gln/Glu ratio. As mentioned, Asn becomes an essential AA upon extracellular Gln depletion, due to its absolute requirement for protein synthesis. Because of this, we measured endogenous levels of Asn and Asp under the same conditions as described in Figure 1. We observed that endogenous Asn levels were reduced significantly upon cGPG depletion and after BPTES and MSO treatment, but not by cPG depletion at normoxia (Figure 3A). Asp, which is a precursor for Asn, was also significantly reduced upon cGPG depletion, however in contrast with Asn, it was not significantly affected by BPTES treatment, and it was increased upon cPG depletion (Figure 3B).

At hypoxia, Asn levels were not significantly affected by either cGPG and cPG depletion nor, BPTES and MSO treatment (Figure 3C). Asp, however, was significantly reduced upon cGPG depletion, increased by cPG depletion and MSO treatment, similar to what was observed at normoxia (Figure 3D). In line with previous reports, T cells could not compensate for Gln depletion by using Asn as a substrate for the TCA cycle, as asparaginase is not expressed in T cells (Supplementary Figure S2) [16]. This indicates that the fluctuation in intracellular Asp is derived from other sources than Asn. In line with this, endogenous Asp levels were also influenced by exogenous cGPG depletion as well as by GS inhibition.

**Figure 4.**
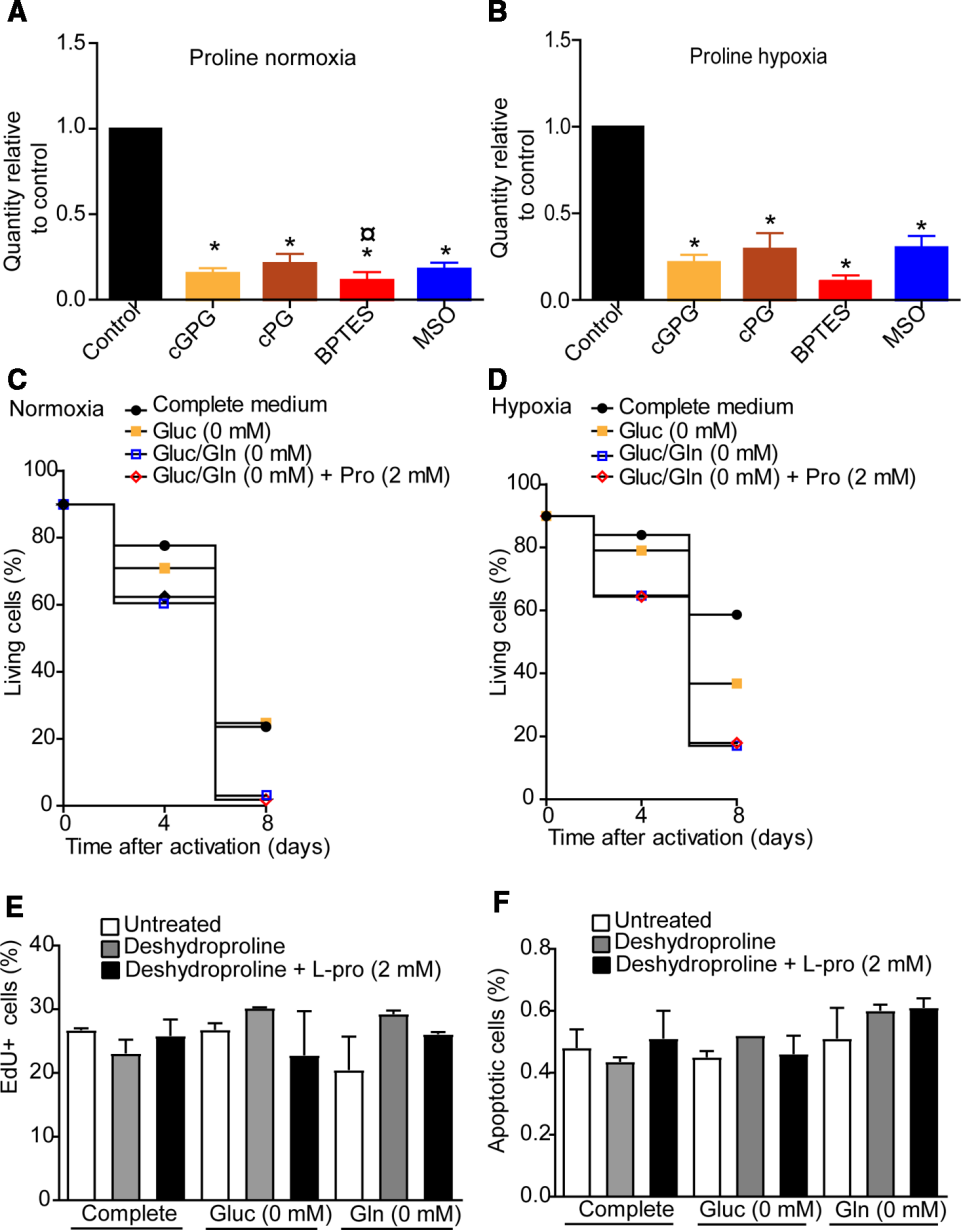
Pro is depleted following Gln deprivation or inhibition of Gln metabolism at normoxia and hypoxia. Relative quantities of Pro at 72 h following 12 h of cGPG or cPG depletion, or BPTES or MSO treatment at normoxia (**A**) and hypoxia (**B**). Proliferation (**C**) and apoptosis (**D**) at 72 h in complete media, gluc-free media or Gln-free media supplemented in the presence or absence of deshydroproline or deshydroproline +2 mM of Pro at normoxia. (**D**) Long-term survival in complete media, gluc-free media or Gln-free media supplemented in the presence or absence of deshydroproline or deshydroproline +2 mM of Pro at normoxia. (**E**) Relative quantities of Pro at 72 h following 12 h of cGPG or cPG depletion, or BPTES or MSO treatment at hypoxia. (**F**) Long-term survival in complete media, gluc-free media or Gln-free media supplemented in the presence or absence of deshydroproline or deshydroproline +2 mM of Pro at hypoxia. Data are mean ± SEM of three independent experiments in triplicates. * *P* < 0.05 compared with control, # *P* < 0.05 compared with cGPG, ¤ *P* < 0.05 compared with cPG, + *P* < 0.05 compared with BPTES.

Pro can be metabolized to, or synthesized from Glu [15]. Pro was significantly reduced upon cGPG and cPG depletion and after BPTES and MSO treatment at both normoxia and hypoxia (Figure 4A,B). It is reported by Geiger and coworkers, that Arg can be metabolized into ornithine to support Pro synthesis [25]. Despite this, Arg levels were only modestly affected by BPTES and MSO treatment at normoxia, while ornithine levels were not (Supplementary Figure S3A,B). At hypoxia, Arg levels were reduced by all treatments, but again, ornithine was not significantly affected (Supplementary Figure S3C,D). Despite this, Pro supplementation could not reverse reduced viability after 8 days of Gluc nor Gln starvation, neither at normoxia, nor at hypoxia (Figure 4C,E). This is in line with previous reports demonstrating that CD4+ T cells rely on endogenous Pro synthesis rather than Pro uptake [25].

It has been shown that Pro has two distinct fates in eukaryotic cells. One is to support collagen synthesis in the cytoplasm and the other is to support energy and carbon substrates through Gln synthesis in the mitochondrion [34]. In short, in the mitochondrion, Pro is converted to 1-pyrroline-5-carboxylic acid (P5C) by the enzyme Pro oxidase (PRODH1) and further via glutamic-γ-semialdehyde (GSA) to Gln via P5C dehydrogenase (P5CDH). To this end, the Pro analog deshydroproline inhibits PRODH1 and prevents the conversion of Pro into P5C [35]. Based on this, the observation that Pro alone could not rescue viability upon Gln deprivation, we tested if extracellular Pro combined with deshydroproline could rescue CD4^+^ T-cell viability under nutrient restricted conditions and at normoxia and hypoxia. Neither proliferation nor apoptosis was significantly altered at 72 h in the presence of exogenous deshydroproline, alone and in conjunction with Pro at normoxia (Figure 4E,F). The same trend was also observed in medium depleted of either Gluc or Gln (Figure 4E,F).

### GLS inhibition reduces glycolysis through reduced HK activity

**Figure 5.**
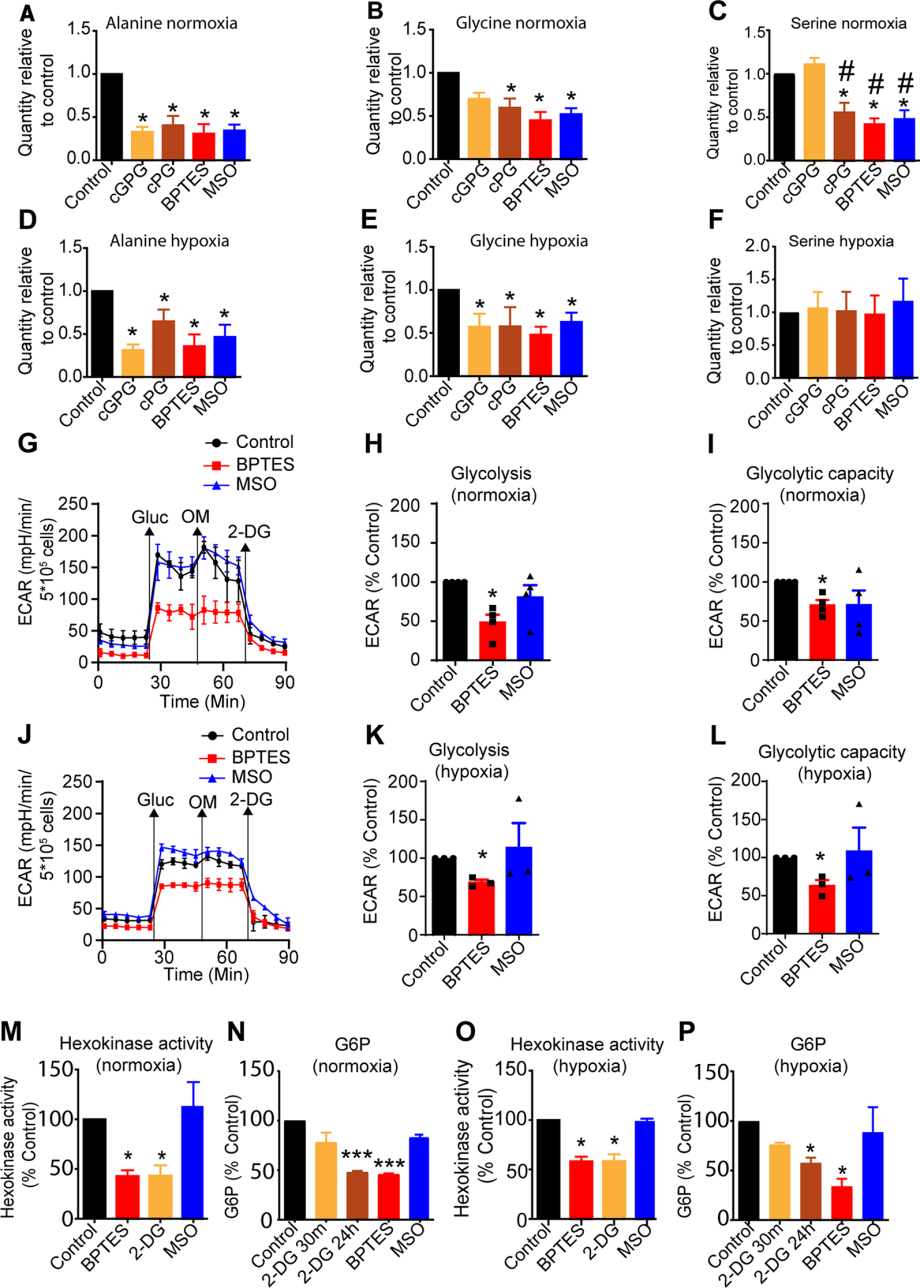
GLS inhibition, but not GS inhibition reduces glycolysis. Relative quantities of Ala (**A**) Gly (**B**) and Ser (**C**) at normoxia and Ala (**D**), Gly (**E**) and Ser (**F**) at hypoxia at 72 h following 12 h of cGPG or cPG depletion, or BPTES or MSO treatment at normoxia. (**G**) Representative Seahorse, glycolytic stress test of control, BPTES and MSO treated cells at normoxia. Glycolysis, (**H**) glycolytic capacity, (**I**) relative to control at normoxia. (**J**) Representative Seahorse experiment of control, BPTES and MSO treated cells at hypoxia. Glycolysis (**K**) and glycolytic capacity (**L**) relative to control at hypoxia. (**M**) HK activity and (**N**) G6P concentration in BPTES, 2-DG and MSO treated cells relative to control at normoxia. (**O**) HK activity and G6P (**P**) concentration in BPTES, MSO and 2-DG treated cells relative to control at hypoxia. Data are mean ± SEM of three independent experiments. * *P* < 0.05, *** *P* < 0.001 compared with control.

We next measured endogenous levels of Ala, Ser and Gly at normoxia and hypoxia. This demonstrated that Ala, Ser but not Gly at normoxia were consistently reduced upon cPG depletion and after BPTES and MSO treatment at normoxia. After cGPG depletion, however, Gly and Ser levels were not altered (Figure 5A–C).

At hypoxia (Figure 5D–F), both Ala and Gly but not Ser were reduced upon cGPG and cPG depletion and after BPTES and MSO treatment (Figure 5F). The three carbon AAs Ala, Ser and Gly may be derived from Pyr and hence glycolytic activity [29,36–38]. Down-regulated glycolytic capacity may involve endogenous Ser levels as glycolytic 3PG is a substrate for the Ser biosynthesis pathway. As the ser biosynthesis pathway is regulated by negative feedback, Ser accumulation increases glycolytic flux [29]. To this end, *de novo* synthesis of Ser and Gly is regulated by glycolytic flux, thus we tested whether changes in Ala, Ser and Gly were related to changes in glycolytic activity.

Glycolytic activity was measured using ECAR employing the Seahorse glycolysis stress test. This demonstrated that at both normoxia and hypoxia (Figure 5G–L), BPTES treatment was associated with down-regulated glycolysis and glycolytic capacity measured as reduced ECAR. As HK activity is reported to correlate with ECAR in activated T cells we next determined if the effect on ECAR was due to altered HK activity [39]. HK activity in CD4^+^ T cells was measured in the presence and absence of 2-DG, BPTES and MSO, both at normoxia and hypoxia. This revealed that GLS, but not GS inhibition down-regulated HK enzyme activity to the same extent as 2-DG, which is a Gluc competitive inhibitor for HK at normoxia (Figure 5M), this was verified by a significant reduction in the level of G6P (Figure 5N). At hypoxia, GLS inhibition also reduced HK activity and 6GP similar to 2-DG (Figure 5O,P). The effect of inhibiting GLS on HK activity and the effect on G6P levels, were verified using the two GLS inhibitors 5-(3-Bromo-4-(dimethylamino)phenyl)-2,2-dimethyl-2,3,5,6-tetrahydrobenzo[a] phenanthridin-4(1H)-one (968), and N-[2-(1,3-Benzothiazol-2-ylamino)ethyl]-2-thioxo-1,2-dihydro-3-pyridinecarboxamide (C19, Enamine, Kyiv, Ukraine) which was recently reported (Supplementary Figure S4A,B) [40]. Based on this we speculated and tested if Gln accumulation by inhibiting GLS would influence HK activity either directly or indirectly. Accordingly, the effect of incremental concentrations of Gln on recombinant HK1 and HK2 enzyme activity was measured. This revealed that Gln at any concentration tested, did not influence neither HK1 nor HK2 activity *in vitro,* concluding that Gln does not directly regulate HK activity (Supplementary Figure S4C,D).

For the complete list of metabolites analyzed see Supplementary Figures S5 and S6.

## Discussion

Adaptation to oxygen and nutrient availability are central requirements to immune cell function and viability. For instance, at both normoxia and hypoxia a number of metabolic challenges and adaptions must occur to sustain optimal immune responses. Within this context, T cells are highly migratory cells of the adaptive immune system that frequently encounters a wide range of oxygen tensions in both health and disease [1,41,42]. Moreover, T cells residing in various parts of the body are also faced with variable availability of key nutrients required for proliferation and survival, such as Gluc and Gln. To this end, responses to environmental changes by immune cells are in large dependent on metabolic flexibility, and it is now clear that oxygen and nutrient availability regulates T-cell differentiation and function [43]. Recently it has been demonstrated that this in part is orchestrated by the expression of hypoxia-inducible factors, which regulate genes defining distinct characteristics of both CD4^+^ and CD8^+^ T-cell subsets [41,44,45]. Here, we have investigated the anabolic and catabolic contributions of Gln in CD4^+^ T cells stimulated through the CD3 and CD28 cell surface markers. Levels of the exogenous nutrients Gluc, Gln and Pyr were depleted as well as the endogenous activity of GLS or GS manipulated at normoxia and hypoxia. Overall, we observed that CD4^+^ T-cell proliferation and viability were more dependent on Gln than Gluc and Pyr. We have previously shown that GLS inhibition reduces proliferation, without inducing cell death, under the same conditions as used in the present study [22]. Kono and coworkers demonstrated that GLS inhibition reduced Th17, but not Th1, Th2 or regulatory T-cell (Treg) differentiation [46]. Moreover, during activation by anti-CD3/CD28, we have previously shown that GLS inhibition downrefulates a number of cytokines associated with various subtypes of CD4+ T cells after 3 days. This indicates overall reduced activation when inhibiting GLS rather than affecting differentiation. However, 5 days post GLS inhibition was associated with increased secretion of IL-10 at hypoxia, but not normoxia. This may be indicative of effects on differentiation as well, together pointing to a complex endogenous metabolic picture regulated by the activity of GLS [22]: We further demonstrated that Gln dependence was higher at hypoxia compared with normoxia. This was supported by the observation that only maximal OCR was sensitive to GLS inhibition under normoxia whereas under hypoxia inhibition of either GLS or GS reduced both basal OCR as well as maximal OCR. Moreover, at hypoxia inhibition of GS also reduced OCR, while this was unaffected at normoxia. This demonstrated that Gln metabolism is differentially regulated at normoxia and hypoxia, suggesting that endogenous Gln synthesis from Glu is rate-limiting under hypoxia. The requirement for GS-dependent production of Gln during hypoxia is in line with our previous report demonstrating increased induction of GS expression under hypoxic conditions in CD4^+^ T cells [22].

Glu required for Gln production may be derived from several substrates. These include the AA Asp, which may support endogenous Gln via the TCA cycle intermediate oxaloacetate and α-KG. The latter is a key intermediate for Glu synthesis through transamination. Glu may also be produced by Pro and Arg [47–50]. From our experiments, we observed that the endogenous levels of Asn, Asp, Pro and Arg, were all differentially regulated during exogenous deprivation of Gln and manipulation of Gln metabolism at normoxia and hypoxia. Although being crucial for T-cell survival in anti-tumor responses, Arg did not appear to be rate-limiting for T-cell proliferation under Gln starvation [25]. Instead, endogenous Pro levels were nearly exhausted under all conditions of nutrient deprivation as well as when GLS and GS were inhibited. However, as exogenous substitution of Pro, even when non-collagen metabolism of Pro was blocked, could not rescue proliferation or viability, this indicates a crucial role for *de novo* synthesis of Pro. This is in line with the reports that Gln may account for up to 80% of endogenous Pro and that T cells are nearly devoid of Pro transporters, and hence will not have the capacity of rescuing endogenous Pro levels with exogenous uptake [14,25,51,52]. This supports a rate-limiting role by endogenous Pro for Gln synthesis and CD4^+^ T-cell proliferation under the acute 12 h deprivation of nutrients investigated here. The mechanism by which Pro abrogates cellular processes such as proliferation and survival requires further investigation but may involve abrogated protein synthesis and reduced production of endogenous Gln.

As shown by Pavlova et al. [16] Asn becomes an essential amino acid when exogenous Gln is depleted. In the same study, they also showed that supplementing Asn to Gln-starved cells partially rescued proliferation, despite that Asn is not contributing to the TCA cycle or Gln synthesis [16]. This is consistent with our results showing that asparaginase is not expressed in T cells. In CD8^+^ T cells, Asn is reported to enhance activation through directly interacting with lymphocyte-specific protein tyrosine kinase (LCK) [53]. Upon 12 h of exogenous cGPG starvation, we observed that Asn was only moderately reduced at normoxia and unaltered at hypoxia. It is reported that Asn is important for maintaining cell viability in Gln-deprived cells, despite not contributing to the TCA cycle via anaplerosis, but is used for protein synthesis [14–16,23]. This suggests that the moderate consumption of endogenous Asn under Gluc and Gln starvation as well as under GLS and GS inhibition is reflected in the rate of proliferation and cell viability. Finally, Asp was completely exhausted under cGPC deprivation whereas it accumulated under cGP deprivation and in the presence of MSO. Based on this we speculate that Asp serves two roles under CD4^+^ T-cell proliferation, anapleurosis for the TCA metabolites and as a substrate for Asn used for protein synthesis.

The 3-carbon AAs, Ala, Ser and Gly are closely linked to glycolysis and may be shunted into glycolysis and the TCA cycle through 2-phosphoglycerate (2-PG), Pyr and Acetyl coenzyme A (AcCoA) [38,54–56]. *De novo* synthesis of Ser and Gly can also fuel purine synthesis through the one-carbon pathway and fuel GSH synthesis [54,57,58]. Furthermore, glycolytic intermediates also serve as substrates for the synthesis of these AAs. Ala may be produced by transamination of Pyr using Glu or Ser as the amino group donator. Ser is produced from 3-PG, and further converted to Gly [25,37,55,56,58]. As uptake of Ala and Gly is negligible, CD4^+^ T cells rely on *de novo* synthesis of Ala and Gly [25]. Pyr can be produced from Gln through the TCA intermediate malate or indirectly through Asp using the Lac /malate/Asp shuttle [54,59,60].

Under both cGPG deprivation and when Gln metabolism was inhibited endogenous Ala, Ser and Gly were differentially regulated under normoxia. This was also the case under hypoxia for Ala and Gly, but not Ser. We interpret that Ala, Ser and Gly compensate for the lack of Pyr produced by glycolysis during Gluc and Pyr deprivation at normoxia. However, since endogenous Ala and Gly levels were reduced also when Gln was depleted and when both GLS and GS were inhibited we speculated that endogenous Gln levels might influence glycolytic activity and Pyr production at normoxia. In line with this, we observed that GLS inhibition and accumulation of endogenous Gln were associated with down-regulated ECAR and decreased HK activity, resulting in reduced levels of G6P. The latter was not due to a direct effect of Gln on HK activity, nor due to off-target effect of BPTES as we could confirm that GLS inhibition using other GLS-specific inhibitors also prevented G6P formation. Rather our observation supported the finding by others who demonstrated that Gln-dependent anaplerosis dictates glucose uptake and cell growth in a variety of proliferating cells including cancer cells [26]. We further showed that HK activity was down-regulated by BPTES at normoxia and hypoxia suggesting that a link between mitochondrial Gln metabolism and glycolysis also exists in CD4^+^ T cells. However, the observations that BPTES reduced glycolysis in hypoxia, despite not affecting levels of Ser, it appears that the BPTES-induced reduction in glycolysis is independent of Ser metabolism. This is supported by the observation that GLS inhibition reduced HK activity and G6P formation, which is upstream of the Ser biosynthesis pathway [58].

Taken together, we observed that oxygen levels together with Gln availability and metabolism, differentially regulate CD4^+^ T-cell proliferation, OCR and glycolysis. Moreover, depletion of exogenous Gln and inhibition of its endogenous metabolism, was acutely associated with Asp and Pro exhaustion, and down-regulation of glycolysis, irrespective of oxygen availability above 4%. This implicates Gln as the key exogenous nutrient required for optimal CD4^+^ T-cell activity and immune activity.

## Materials and methods

### CD4^+^ T cell isolation, culture and stimulation

Human CD4^+^ T cells were isolated from buffy coats of healthy donors (supplied by Blodbanken, Oslo, Norway) using the Dynabeads CD4 Positive Isolation kit (Life Technologies AS, Oslo Norway) as previously described [22]. Buffy coats were diluted 1 : 2 with RPMI 1640 (Sigma–Aldrich, St. Louis, MO, U.S.A.) and 1 ml EDTA and rotated for 15 min at 4°C. CD4^+^ T cells were then isolated according to the company protocol. Cells were maintained at 5 × 10^6^ cells/ml unless otherwise stated. CD4^+^ T-cell activation was initiated by adding pre-washed Human T-Activator CD3/CD28 Dynabeads at a 1 : 4 bead/cells ratio (Life Technologies).

### H^3^-Thymidine incorporation

Isolated CD4^+^ T cells were stimulated and cultured in DMEM 1× (no glucose, no glutamine) with 5% NaHCO_3_, 10% dialyzed FBS, 14 mM d-glucose or RPMI 1640 (Sigma–Aldrich) supplemented with 10% heat inactivated FBS (Sigma–Aldrich); 2 mM glutamine (Sigma–Aldrich) 0.5% Penicillin-Streptomycin (Sigma–Aldrich). 5 × 10^6^ T cells were incubated in 150 μl of media under hypoxia (O_2_ = 1%) or normoxia (O_2_ = 21%) for 72 h during which H^3^-thymidine (1 μCi/well; PerkinElmer, Boston, MA, U.S.A.) was added to the media for the last 12 h of incubation. H^3^-thymidine incorporation on the 72nd hour was counted using a Wallac MicroBeta Counter (PerkinElmer).

### Flow cytometry

Proliferation was measured using 5-ethynyl-2′-deoxyuridine (EdU) iFluor 488 Proliferation kit according to manufacturer's instructions (Abcam, ab219801). Briefly, anti-CD3/CD28 cells were cultured for a total of 72 h. In the last 12 h, cells were diluted to a concentration of 10^6^ cells/ml in complete medium or medium depleted of either Gluc or Gln in the presence or absence of deshydroproline or deshydroproline + Pro, 20 µM EdU was added in the final 2 h. Cells were collected and centrifuged, then washed twice in wash buffer before being fixed by adding 4% paraformaldehyde for 15 min followed by centrifugation then resuspended in permeabilization buffer for 15 min before adding the EdU reaction mix to each tube followed by 30 min incubation at room temperature in the dark. Cells were then washed twice and resuspended in PBS before data was acquired using FACS Canto II and analyzed using Flow-Jo v10 Stoftware (Tree Star, Ashland, OR, U.S.A.).

To detect cell death, cells were stained with Dead Cell Apoptosis Kit with Annexin V FITC and PI according to the user manual (BD BioSciences Franklin Lakes, NJ, U.S.A.). Briefly, cells were harvested, washed in cold PBS then resuspended in 1× Annexin binding buffer at a concentration of 1 × 10^6^ cells per ml. 1 × 10^5^ cells in 100 µl were incubated with 5 µl of Annexin V and 5 µl PI for 15 min in the dark. After incubation cells were diluted to 500 µl in Annexin V binding buffer and data was acquired with the FACS Canto II and analyzed using Flow-Jo V10 software (Tree Star, Ashland, OR, U.S.A.).

### Bioenergetic measurements

Oxygen consumption rates (OCR) and extracellular acidification rates (ECAR) were measured using Extracellular XF24e Flux Analyzer (Seahorse Bioscience North Billerica, MA, U.S.A.). OCR of 5 × 10^5^ CD4^+^ T cells were measured in non-buffered RPMI 1640 containing 10 mM glucose, 2 mM L-glutamine and 1 mM sodium pyruvate (Seahorse Bioscience) under basal conditions and in response to 1 μM oligomycin (OM), 1.5 μM Carbonyl cyanide *m*-chlorophenyl hydrazone (CCCP) and 100 nM rotenone + 1 μM antimycin A (Sigma) (RA), according to company protocol for the Mito Stress Test (Seahorse Bioscience). Cells were treated with 25 μM Bis-2-(5-phenylacetamido-1,3,4-thiadiazol-2-yl) ethyl sulfide (BPTES, Sigma–Aldrich) and 7.5 mM methionine sulfoximine (MSO, Sigma–Aldrich). Extracellular acidification rate (ECAR) was measured in non-buffered RPMI 1640 containing 2 mM l-glutamine and 32 mM additional sodium chloride under basal conditions in response to 10 mM Glucose, 1 μM OM and 20 mM 2-deoxy-D-Glucose (2-DG) according to the company protocol for Glycolysis Stress Test (Seahorse Bioscience).

### Hexokinase activity

HK activity was determined using hexokinase activity assay. Briefly, 1 × 10^6^ cells were washed in cold PBS then resuspended in 200 µl cold reaction buffer (50 mM Tris HCl and 10 mM MgCl_2_), homogenized and then centrifuged at 12 000rpm at 4°C for 5 min. Supernatants were collected and 50 µl sample or 0.5 µg/50 µl recombinant HK1 or HK2 was transferred to a 96 well plate then mixed with 50 µl of 2× reaction mix (50 mM TRIS HCl, 10 MgCl_2_, 1.2 mM ATP, 200 mM Gluc, 0.4 mM NADPH and 0.2 units/ml Glucose 6-phosphate dehydrogenase from *Saccharomyces cerevisiae*). Plates were incubated for 15 min at room temperature protected from light then OD was measured using Epoch (BioTek, Winooski, VT, U.S.A.) microplate reader at 340 nm every 15 s for 10 min or every 120 s for 60 min.

### Glucose 6-phosphate measurement

G6P was measured according to instructions by Sigma (MAK014). Briefly, cells were homogenized in ice cold PBS, centrifuged at 13 000***g*** for 10 min, and deproteinized using a 10 kDa MWCO Viva Spin column (GE Healthcare, Chicago, IL, U.S.A.). Samples were next diluted in G6P assay buffer and mixed with 50 µl of G6P reaction buffer, and incubated at RT for 30 min. OD was measured using Epoch (BioTek) microplate reader at 450 nm. Samples were normalized to control.

### Metabolic tracing of amino acids

One million cells were incubated in the presence of 0.5 mM ^15^N_2_-^13^C_5_- Glutamine (Sigma–Aldrich) and 5 mM D-glucose (Sigma–Aldrich) in DMEM no glucose, no glutamine medium and collected at 2 h by centrifugation (15 000 rpm, 4°C). Metabolites of interest were extracted immediately by adding 300 µl 80% MeOH (−78°C). Cell debris was sedimented at 15 000 rpm, 4°C for 15 min. Subsequently, supernatants were dried using a speed vac (DNA100 Speed Vac, Thermo Scientific™ Savant™, Waltham, MA, U.S.A.) and stored at −78°C before being analyzed. For liquid-chromatography–tandem mass spectrometry (LC/MS–MS) analysis the samples were diluted in a mobile phase of 0.5% Formic acid (FA), 0.3% Heptafluorobutyric acid (HFBA). The labeled ^15^N_2_-^13^C_5_-Gln and ^15^N-^13^C_5_- glutamate were separated isocratically by retention using a Pheomenex Kinetex core shell C18 (100 × 4.6 mm, 2.6 µm) column. The amounts of ^15^N_2_-^13^C_5_-Gln were determined relative to the internal standard by integrating the area of each chromatographic peak. The concentrations of AA in cells were analyzed by liquid chromatography–tandem mass spectrometry (LC–MS/MS) using modifications of a previously described method [61]. Briefly, prior to analysis the cell samples were resolved in 20 µl water. Then, 20 µl of cell samples, calibration standards, quality control samples (human plasma), and blank matrix (water) were added to a 1 ml 96 well extraction plate. Deuterated internal standard mix of all AA (10 µl) was added to all wells. We further added 10 µl of ammonium solution [10 mM] to neutralize the samples, before adding 10 µl of 100 mM 1,4 dithioerythritol (DTT) to reduce cystine and cysteine mixed disulfides into reduced cysteine. The solutions in the wells were gently mixed using a plate shaker at room temperature for 15 min. Then 10 µl 5-sulfosalicyclic acid (SSA) was added to precipitate plasma proteins. Subsequently, samples were mixed using a plate shaker and centrifuged at 4000 rpm for 15 min. Supernatant (15 µl) from the extraction plate diluted in 135 μl mobile phase A in a shallow 96 well analysis plate.

Extracted AA were analyzed using 5500 Q TRAP linear MS/MS spectrometer tandem mass spectrometer (AB Sciex, Framingham, MA). The chromatographic separation was performed on a Phenomenex Kinetex Core Shell C18 (100 × 4.6 mm, 2.6 µm) HPLC column (Phenomenex Torrance, CA, U.S.A. and thermostated at 30°C. The mobile phases were (A) water/formic acid (100 : 0.05, v/v) and (B) acetonitrile /formic acid (100 : 0.05, v/v) at a flow rate of 0.2 ml/min. The separation was achieved with a linear gradient from 100% (A) for 2 min, 40% (A) from 2 to 6 min followed by a linear gradient back to 100% (A) over 2 min. The whole run was 6.5 min, and the injection volume was 20 µl. We then identified each amino acid by MS, corresponding to each particular internal standard. We determined the concentration of each amino acid from the ratio of analyte peak area/internal standard peak area against a linear multiple point calibration curve

### Protein gel separation and Western blot

Cells were pelleted by centrifugation then washed with cold PBS, followed by lysis in Radioimmunoprecipitation assay (RIPA) buffer containing inhibitors of proteases (Sigma P5726, 1 : 100) and phosphatases (Sigma P8340, 1 : 100). 4× Laemmli buffer (Bio-Rad, Hercules, CA, U.S.A.) was added and samples heated to 95°C for 5 min. An amount of 40 µg protein was loaded in each well in a 15-well 10% polyacrylamide mini-PROTEAN TGX precast Tris-glycine gel (Bio-Rad). Separated proteins were transferred to a nitrocellulose membrane using the Trans-blot Turbo transfer system using the program ‘mixed MW’. Following transfer, membranes were first blocked with 5% no-fat milk (Bio-Rad) in Tris-buffered saline with 0.01% Tween 20, pH 7.4 (TBST) for 30 min. Next, membranes were incubated with primary antibodies overnight (4°C) diluted in 1% no-fat milk in TBST, followed by washing with TBST and finally incubation with HRP-conjugated secondary antibodies for 2 h. Substrate (SuperSignal™ West Dura Extended Duration Substrate, Thermo Fisher Scientific) was added to the membrane and product formation detected using ChemiDoc XRS+ system (Bio-Rad) and Image Lab 4.1 (Bio-Rad). Membranes were stripped using Restore PLUS western blot stripping buffer (Thermo Fisher Scientific) according to manufacturer's instructions, then blocked and stained with appropriate antibodies. Anti-L-asparaginase (polyclonal #65552, 1 : 1000), anti-GAPDH (rabbit monoclonal clone 14C10. 1 : 1000) and HRP-conjugated rabbit IgG (polyclonal #7074) were purchased Cell Signaling Technology (Danvers, MA, U.S.A.). ASRGL1 overexpression lysate (NBP2-06243) was purchased from Novus Biologicals (Centennial, U.S.A.) to be used as positive control for human l-asparaginase.

### Statistics

Statistical analysis has been performed using GraphPad Prism 6 (La Jolla, CA, U.S.A.). Results were analyzed by two-way ANOVA to compare multiple groups, or student's *t*- test to compare paired groups.

## Data Availability

The authors declare that all data supporting the findings and conclusions of this study are available within the article or Supplementary Figures.

## Abbreviations

2-DG: 2-deoxy-D-Glucose
AA: amino acid
ALT: Ala transaminase
BPTES: Bis-2-(5-phenylacetamido-1,3,4-thiadiazol-2-yl) ethyl sulfide
ECAR: extracellular acidification rate
GLS: glutaminase
GS: glutamine synthase
GSH: glutathione
HK: hexokinase
α-KG: alpha-ketoglutarate
MSO: methionine sulfoximine
NADPH: Nicotinamide adenine dinucleotide phosphate
OCR: oxygen consumption rate
OM: oligomycin
TCA: tricarboxylic acid
