# Supplementary material for: Endogenous glutamine is rate-limiting for anti-CD3 and anti-CD28 induced CD4+ T-cell proliferation and glycolytic activity under hypoxia and normoxia
Source: Biochem J. 2022 Jun 13;479(11):1221–35. doi: 10.1042/BCJ20220144 (PMC9246347; doi:10.1042/BCJ20220144)
Supplement: Supplementary Material [file BCJ-479-1221-s1.pdf]

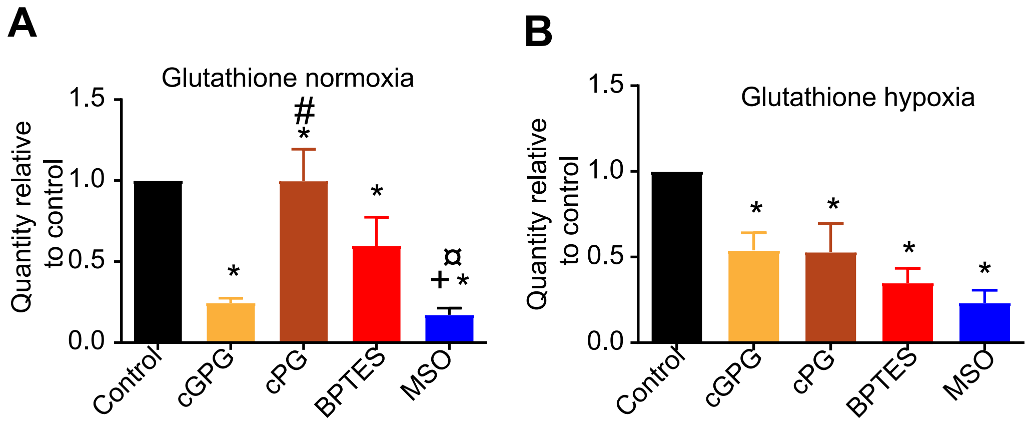

Figure S1 Gln deprivation and inhibition of Gln metabolism reduces GSH concentrations. Relative quantities of GSH at normoxia (A) and hypoxia (B) in $CD4^+$ T cells at 72 hours post anti-CD3/CD28-stimulation following 12 hours of cGPG or cGP depletion or BPTES or MSO treatment. Data are mean ± SEM of 3 independent experiments in triplicates. *p<0.05 and compared to stimulated #p<0.05 compared to cGPG, ¤p<0.05, compared to cPG, +p<0.05 compared to BPTES.

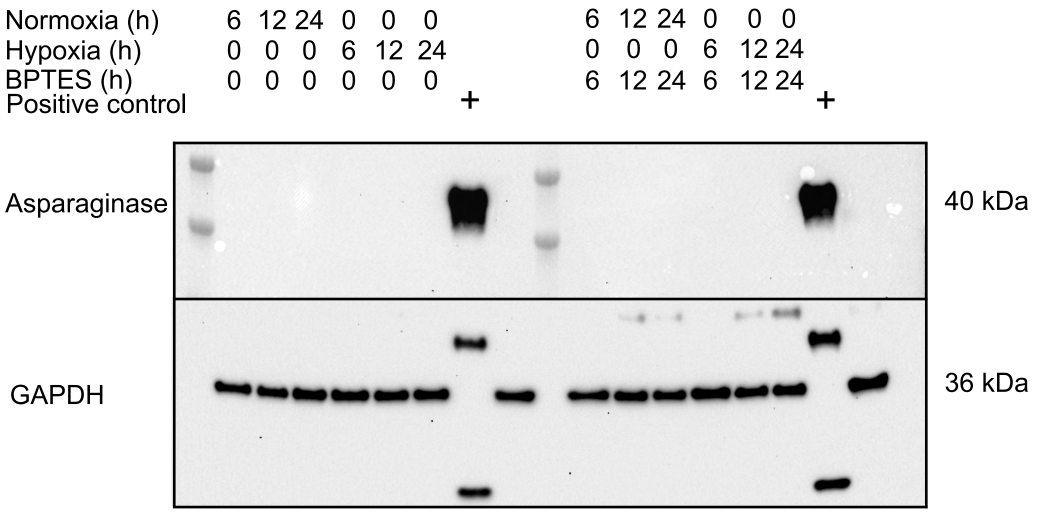

Figure S2 Asparaginase is not expressed in CD4⁺ T cells. Western blot of human asparaginase (Asparaginase-like protein 1) in anti-CD3/CD28-stimulated CD4⁺ T cells following BPTES treatment at normoxia and hypoxia. HEK293 over-expression lysate is used as positive control. Representative of 3 experiments.

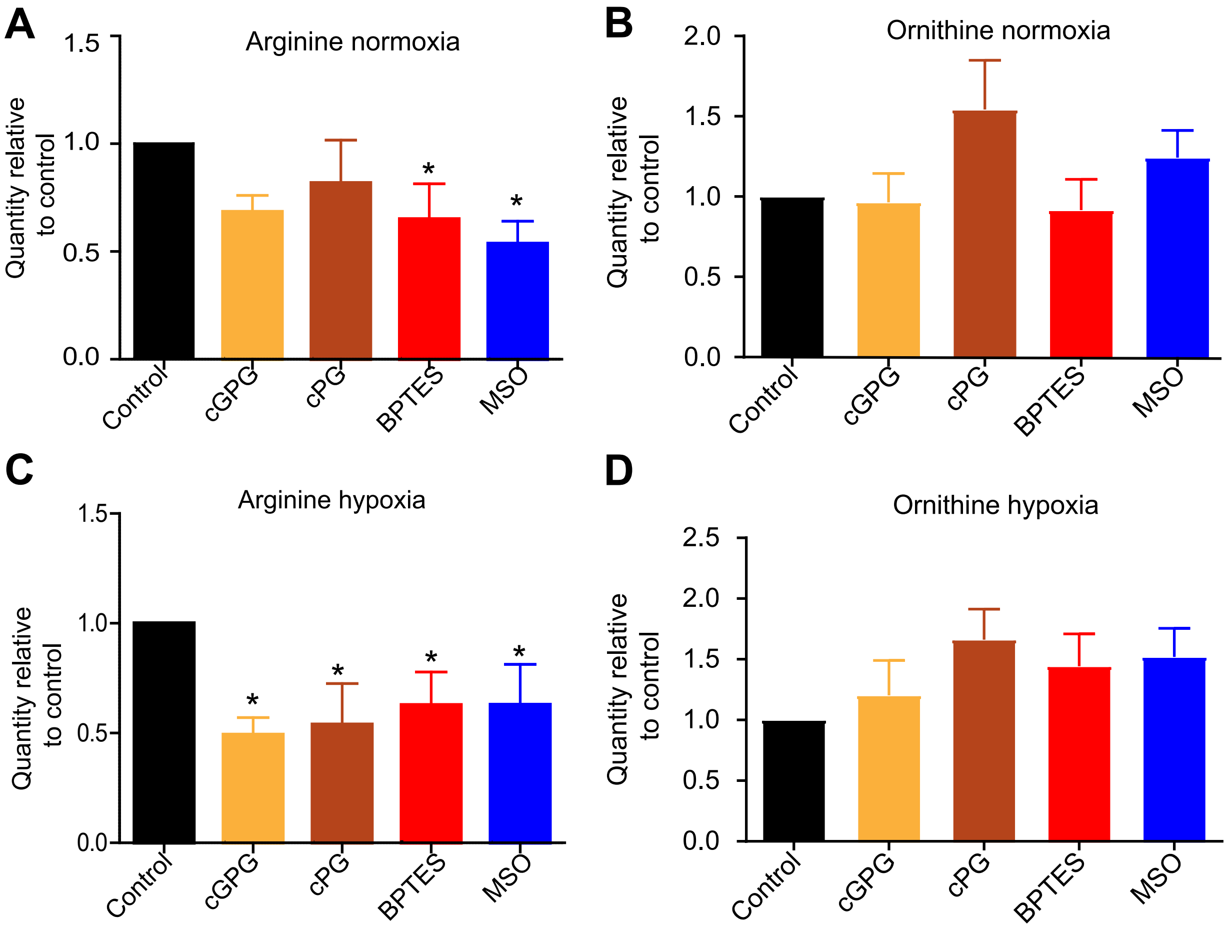

Figure S3 Gln deprivation and inhibition of Gln metabolism differentially affects Arg levels in normoxia and hypoxia. Relative quantities of Arg (A) and ornithine (B) at normoxia and relative quantities of Arg (C) and ornithine (D) at hypoxia in $CD4^{+}$ T cells at 72 hours post anti-CD3/CD28-stimulation following 12 hours of cGPG or cPG depletion or BPTES or MSO treatment. Data are mean ± SEM of 3 independent experiments in triplicates. *p<0.05 compared to control

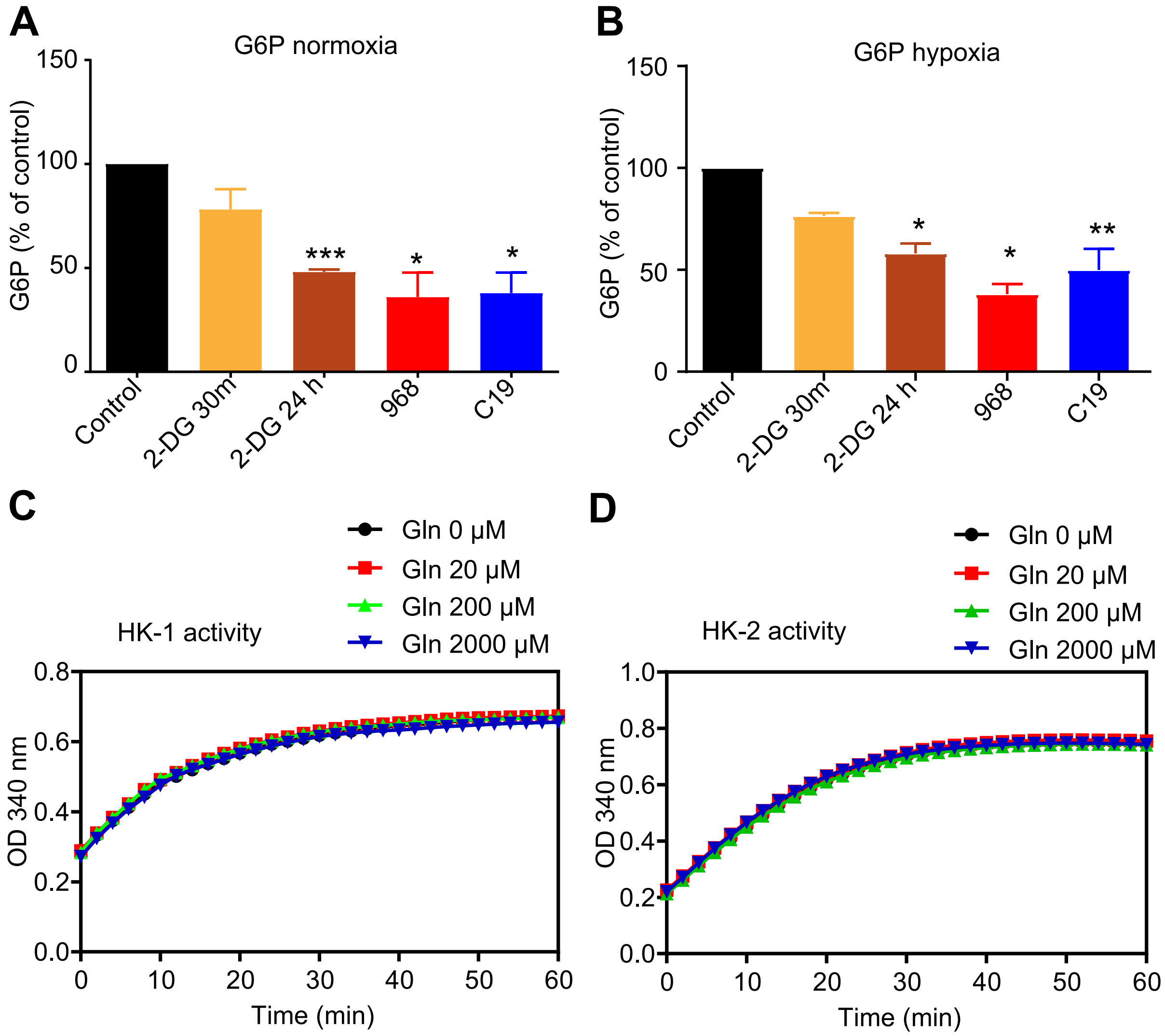

Figure S4 GLS inhibition reduces HK activity independently of Gln accumulation. Relative quantities of G6P in CD4⁺ T cells at 72 hours post anti-CD3/CD28 stimulation following 12 hours of treatment with the GLS inhibitors 968 (40 μM) and C19 (25 μM), or 30 minutes and 24 hours with 2-DG at normoxia (A) and hypoxia (B). Activity of recombinant HK1 (C) and HK2 (D) in the presence of increasing concentrations of Gln. Data are mean ± SEM of 3 independent experiments in triplicates. *p<0.05, **p<0.01 compared to control.

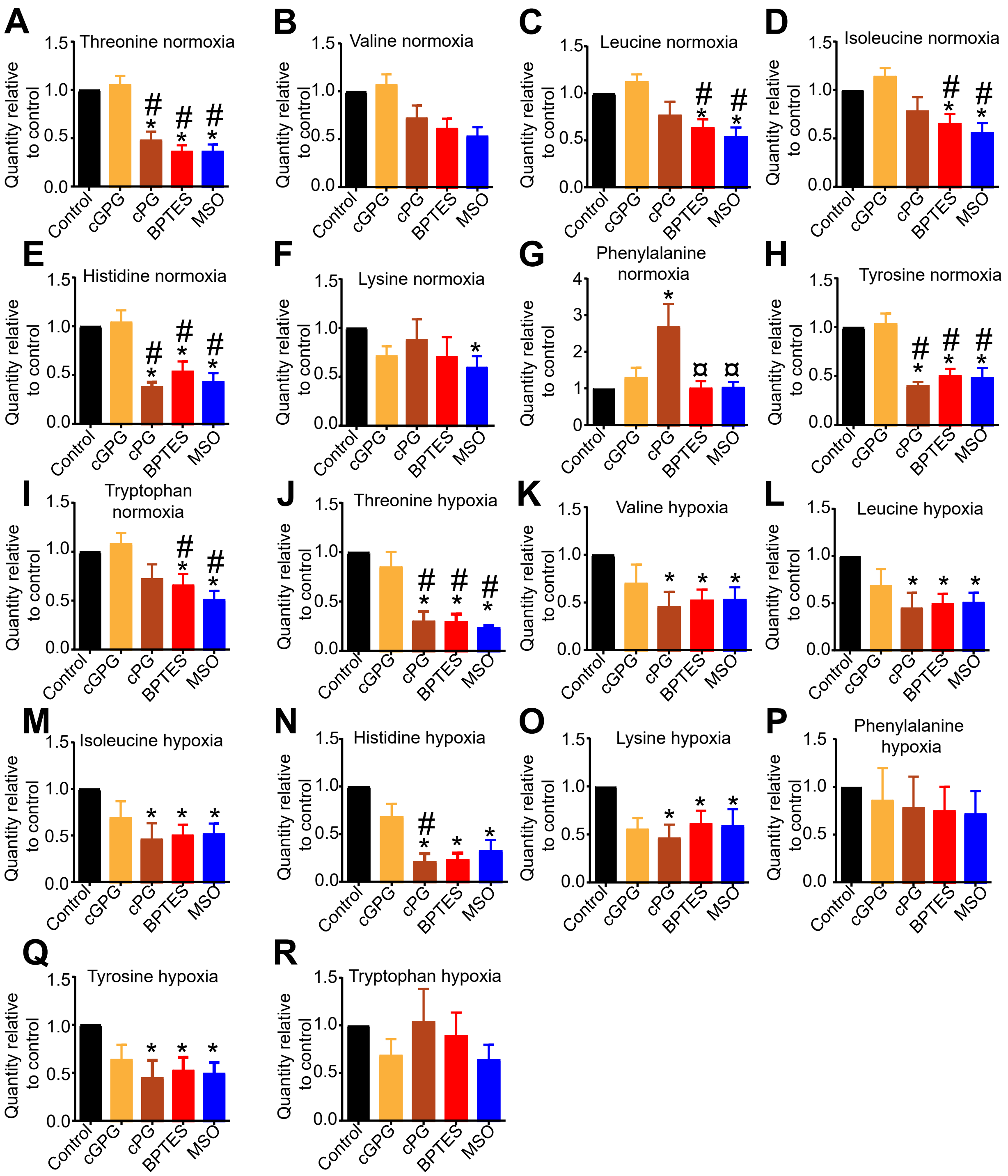

Figure S5 Effect of Gln deprivation and inhibition of Gln metabolism on intra-cellular AA. Relative quantities of threonine (A), valine (B), leucine (C), isoleucine (D), histidine E), lysine (F), phenylalanine (G) tyrosine (H) tryptophan (I) at normoxia and threonine (J), valine (K), leucine (L), isoleucine (M), histidine (N) lysine (O), phenylalanine (P), tyrosine (Q) and tryptophane (R) at hypoxia at 72 hours post anti-CD3/CD28-stimulation following 12 hours of cGPG, cPG depletion or BPTES or MSO treatment in normoxia. Data are mean ± SEM of 3 independent experiments in triplicates. *p<0.05 compared to control, #p<0.05 compared cGPG, ¤p<0.05 compared to cPG.
